# Deoptimization of FMDV P1 Region Results in Robust Serotype-Independent Viral Attenuation

**DOI:** 10.3390/v15061332

**Published:** 2023-06-06

**Authors:** Gisselle N. Medina, Edward Spinard, Paul A. Azzinaro, Monica Rodriguez-Calzada, Joseph Gutkoska, Anna Kloc, Elizabeth A. Rieder, Bruce E. Taillon, Stephen Mueller, Teresa de los Santos, Fayna Diaz-San Segundo

**Affiliations:** 1 Plum Island Animal Disease Center (PIADC), ARS, USDA, Greenport, NY 11944, USA; edward.spinard@usda.gov (E.S.); paul.azzinaro@usda.gov (P.A.A.); monica.rodriguez-calzada@usda.gov (M.R.-C.); teresa.delossantos@usda.gov (T.d.l.S.); fayna.diazsansegundo@nih.gov (F.D.-S.S.); 2 National Bio and Agro-Defense Facility (NBAF), ARS, USDA, Manhattan, KS 66502, USA; 3 ORISE-PIADC Research Participation Program, Oak Ridge, TN 37831, USA; 4 Department of Biology and Environmental Sciences, University of New Haven, West Haven, CT 06516, USA; akloc@newhaven.edu; 5 Codagenix, Inc., Farmingdale, NY 11735, USAmueller@codagenix.com (S.M.); 6 National Institute of Health, NIAID, DMID, OBRRTR, Bethesda, MD 20892, USA

**Keywords:** foot-and-mouth disease, FMDV, vaccines, deoptimization, codon-pair bias, picornavirus

## Abstract

Foot-and-mouth disease (FMD), caused by the FMD virus (FMDV), is a highly contagious disease of cloven-hoofed livestock that can have severe economic impacts. Control and prevention strategies, including the development of improved vaccines, are urgently needed to effectively control FMD outbreaks in endemic settings. Previously, we employed two distinct strategies (codon pair bias deoptimization (CPD) and codon bias deoptimization (CD)) to deoptimize various regions of the FMDV serotype A subtype A12 genome, which resulted in the development of an attenuated virus in vitro and in vivo, inducing varying levels of humoral responses. In the current study, we examined the versatility of the system by using CPD applied to the P1 capsid coding region of FMDV serotype A subtype, A24, and another serotype, Asia1. Viruses carrying recoded P1 (A24-P1Deopt or Asia1-P1Deopt) exhibited different degrees of attenuation (i.e., delayed viral growth kinetics and replication) in cultured cells. Studies in vivo using a mouse model of FMD demonstrated that inoculation with the A24-P1Deopt and Asia1-P1Deopt strains elicited a strong humoral immune response capable of offering protection against challenge with homologous wildtype (WT) viruses. However, different results were obtained in pigs. While clear attenuation was detected for both the A24-P1Deopt and Asia1-P1Deopt strains, only a limited induction of adaptive immunity and protection against challenge was detected, depending on the inoculated dose and serotype deoptimized. Our work demonstrates that while CPD of the P1 coding region attenuates viral strains of multiple FMDV serotypes/subtypes, a thorough assessment of virulence and induction of adaptive immunity in the natural host is required in each case in order to finely adjust the degree of deoptimization required for attenuation without affecting the induction of protective adaptive immune responses.

## 1. Introduction

Foot-and-mouth disease (FMD) is considered one of the most contagious diseases infecting several species of livestock and wild cloven-hoofed animals, including cattle, swine, sheep, goats, and deer [1]. Its etiological agent, the FMD virus (FMDV), belongs to the *Picornaviridae* family and contains a positive-sense single-stranded RNA genome encoding an error-prone RNA polymerase that contributes to FMDV genome diversity. Seven distinct serotypes of FMDV, A, Asia, C, O, and Southern African Territories (SAT) 1, 2, and 3, are recognized, with multiple topotypes and lineages occurring within each serotype [2]. The clinical presentation of FMD is characterized by elevated body temperature and the development of blisters, primarily on the mouth, tongue, and feet, which may lead to lameness and a loss of appetite and weight.

Despite its low morbidity, FMD outbreaks carry a severe economic impact in the countries that report the disease to the World Organization for Animal Health (WOAH, formerly the Office International des Epizooties [OIE]), with an estimated global economic loss between USD 6.5 and 21 billion [3]. Various measures have been implemented to control FMD, including, but not limited to, culling, surveillance, diagnostics, and vaccination. The use of inactivated whole virus vaccines has been instrumental in controlling and, in some cases, eradicating FMD in Western Europe and South America [4,5]. In other parts of the world, FMD remains endemic, and many countries use and store inactivated whole antigen vaccines containing a combination of viruses relevant to the circulating specific serotypes [6]. However, the use of this vaccine is limited due to several factors, such as the need for high-containment facilities for production, the intrinsic vaccine thermolability, and the potential risk of inadequate virus inactivation during production, which may lead to possible release into the environment. In addition, vaccinated animals remain susceptible to FMD if they are exposed to the virus either within 7 days or after approximately 6 months post-vaccination because inactivated vaccines take about a week to induce protective immunity in swine and cattle and the duration of immunity is shorter than that provided by natural infection [7,8]. Thus, there is currently a need to develop safer and more effective novel vaccines. 

Live attenuated vaccines (LAVs) have been shown to induce rapid and long-lasting protection against viral infection and, in some cases, have led to disease eradication (i.e., Rinderpest, reviewed in [9]). Advancements in LAVs for poliovirus, another member of the *Picornaviridae* family, make them an alternative option for FMD vaccines [10]. We previously developed an FMDV LAV candidate by deleting the coding region of L^pro^, a nonstructural viral protease (leaderless virus; [11]). However, this mutant virus was not effective in eliciting a robust adaptive immune response when administered live, and animals were not fully protected against wild-type (WT) virus challenge [12]. Further studies on L^pro^ led to the identification of a conserved protein motif that represents a virulence factor: the SAF-A/B, Acinus, and PIAS (SAP) domain [13]. Substitutions of two conserved amino acid residues in the SAP domain of the A12 strain generated a viral strain that was attenuated in cell culture and in swine. Interestingly, the modified attenuated virus provided complete protection for swine, even when animals were challenged as early as two days post-vaccination [14]. However, due to the limited number of nucleotide changes in the SAP mutant, reversion to virulence still poses a considerable risk, given the high frequency of mutations and recombination that have been described for FMDV in natural hosts [15,16,17,18].

Most recently, the recoding of viral genomes through codon deoptimization has offered an alternative approach for the generation of efficient LAV candidates for different viruses (reviewed in [19]). This approach is based on the natural occurrence of “codon bias”, where codons or pairs of codons are overrepresented while others are underrepresented in protein-coding sequences in specific species (reviewed in [20]). Thus, synonymous mutations can be introduced into viral genomes without altering the encoded protein sequences, which allows the replication of these recoded viruses within the host. Moreover, the incorporation of many nucleotide changes in the viral genome reduces the likelihood of reversion to virulence, thus contributing to the development of safer LAVs.

In proof-of-concept studies, we demonstrated that deoptimization is tolerated by FMDV [21]. In particular, the deoptimization of the P1 structural coding region resulted in an FMDV serotype A (A12-P1Deopt) that was highly attenuated in mice and in swine. In addition, the deoptimization of the P2 and P3 regions, encoding non-structural proteins in the same FMDV serotype A but a different strain, A_24_ Cruzeiro (A_24_Cru), was also well tolerated and resulted in viable progeny with reduced replicative fitness in vivo and in vitro [22]. 

In this study, we have expanded this knowledge by testing whether the deoptimization of P1 could be tolerated in other FMDV serotypes (Asia1 Shamir) or additional FMDV A subtypes (A24). Stable novel viable strains were produced by replacing the P1 coding region of an A_24_Cru infectious clone (IC) [23] with a codon pair-deoptimized P1 region from two FMDV serotypes, A24 and Asia1. Our results indicate that codon pair bias deoptimization (CPD) of the P1 region in these two FMDV strains resulted in phenotypic changes that altered distinct aspects of viral fitness, including viral plaque size, growth kinetics, and replication. Studies in mice and swine demonstrated that the resulting A24-P1Deopt and Asia1-P1Deopt strains were remarkably attenuated in vivo and elicited an adaptive immune response. However, in swine, limited induction of adaptive immunity and protection against challenge were detected, depending on the inoculated dose and serotype deoptimized. These results highlight that (1) CPD targeting the P1 coding region can decrease the virulence of multiple FMDV serotypes and (2) the A_24_Cru IC backbone can be employed to derive recombinant deoptimized viruses of different serotypes by substituting the capsid region of interest. Taken together, our results suggest that deoptimized vaccines could become attractive candidates for further development into modified live attenuated FMDV vaccines.

## 2. Material and Methods

### 2.1. Cells

Porcine kidney cell lines (LF-BKαVβ6) were obtained from the Foreign Animal Disease Diagnostic Laboratory (FADDL) [24], Animal, Plant, and Health Inspection Service (APHIS), at the PIADC. BHK-21 cells (baby hamster kidney cells strain 21, clone 13, ATCC CL10) were obtained from the American Type Culture Collection (ATCC, Rockville, MD, USA). All cells were maintained as previously reported [13]. 

### 2.2. Viruses

FMDV A24-WT was generated from the full-length serotype A_24_ Cruzeiro (A_24_Cru) infectious clone (IC) (pA_24_-WT) as previously described [23]. Challenge virus stocks for FMDV Asia1-Shamir-ISR/89 were derived as previously described [25]. A derivative of this plasmid was constructed to contain an *FseI* unique site at the beginning of VP4 and an *NheI* unique site in the 2A coding region (pA24 Cru-*FseI-NheI*). Deoptimized P1 for FMDV A24 and Asia1 Shamir sequences were created by using the SAVE method for deoptimization [26] and included *FseI-NheI* sites for easy P1 exchange in the pA24Cru-*FseI/NheI* infectious clone. Plasmids were linearized with *SwaI,* and viral RNA was derived through in vitro transcription with T7 polymerase using a MEGAscript T7 kit (Ambion-ThermoFisher Scientific, Waltham, MA, USA) then subsequently treated with DNase and purified with an RNeasy kit (Qiagen) following the manufacturer’s directions. Next, 10–20 ug of viral RNAs was electroporated in BHK-21 cells as previously described [23], and after 24 h incubation at 37 °C, cells were frozen for subsequent virus release and passage. Recovered viruses were sequenced and used for large-scale preparation. Viruses used in this study had been passaged in cell culture at least 10 times. Virus stocks were purified and concentrated through density gradient centrifugation in sucrose 10–50% (*w*/*v*). 

### 2.3. FMDV Cell Infections

Cultured cell monolayers were infected with FMDV at a multiplicity of infection (MOI) of 5. After 1 h adsorption at 37 °C, unabsorbed virus was removed by washing the cells with a solution containing 150 mM NaCl in 20 mM morpholine ethane sulfonic acid (MES) pH = 5.5, before adding MEM and proceeding with incubation at 37 °C in 5% CO_2_. Supernatants from infected cells were collected and frozen at 1, 2, 4, 7, and 24 h, and virus titers were determined through plaque assay on BHK-21 cells. Plaques were counted at 48 h post-inoculation (hpi). 

### 2.4. Western Blotting

Cellular lysates were prepared as described previously [27]. Proteins were analyzed through Western blotting using the following antibodies: anti-eIF4G polyclonal antibody from Bethyl Laboratories (Montgomery, TX, USA) and anti-tubulin monoclonal antibody from NeoMarkers (Fremont, CA, USA). The VP1 and L protease (L^pro^) signal was detected using a monoclonal antibody developed at the Plum Island Animal Disease Center (PIADC). Goat anti-rabbit immunoglobulin G (IgG) and goat anti-mouse IgG secondary antibodies conjugated to the enzyme horseradish peroxidase (HRP) were obtained from Pierce (Rockford, IL, USA). Protein samples were separated through SDS-PAGE and detected through WB using the specific antibodies indicated above and an ECL chemiluminescence kit (Bio-Rad, Hercules, CA, USA). Images were acquired with the chemiluminescence digital imager Azure R Imager c300 (Azure Biosystems, Dublin, CA, USA).

### 2.5. Animal Experiments

Animal experiments were performed in the high-containment facilities of PIADC and conducted in compliance with the Animal Welfare Act (AWA), the 2011 Guide for Care and Use of Laboratory Animals, the 2002 PHS Policy for the Humane Care and Use of Laboratory Animals, and the U.S. Government Principles for Utilization and Care of Vertebrate Animals Used in Testing, Research and Training (IRAC 1985), as well as specific animal protocols reviewed and approved by the Institutional Animal Care and Use Committee (IACUC) of PIADC (USDA/APHIS/AC Certificate number: 21-F-0001; Protocol 204–14R for mice and 151–13R for swine). 

### 2.6. Mice Experiment

C57BL/6 6–7-week-old female mice were purchased from Jackson Labs (Bar Harbor, ME, USA) and were acclimated for one week. Groups of mice (n = 6) were infected with Asia1 Shamir WT subcutaneously (SQ) in the left rear footpad (50 μL) with different amounts of FMDV Asia1 Shamir WT to determine the minimum lethal dose 100. Mice were anesthetized with isoflurane (Webster Veterinary, Devens, MA, USA) and immediately infected SQ in the left rear footpad with 50–100 μL of different doses of FMDV Asia1 Shamir WT. Animals were monitored for 8 days. Serum samples were collected every other day to check viremia through plaque assay or end-point dilution on BHK-21 cells. Two experiments to evaluate the virulence of deoptimized viruses were performed: (1) comparison of A24-P1Deopt vs. A24 WT; (2) comparison of Asia1-P1Deopt vs. Asia1 WT. Animals were monitored and sampled as indicated above. After the first week, serum samples were collected weekly to assess neutralizing antibody responses. Animals surviving the initial inoculation with live attenuated viruses were challenged SQ in the right rear footpad with a lethal dose of homologous WT virus and monitored and sampled as indicated above.

### 2.7. Swine Experiments

Two experiments to evaluate virulence in the natural host were performed. In the first experiment, 20 Yorkshire gilts (five weeks old and weighing approximately 18–23 kg each) were divided into 5 groups of 4 animals each and acclimated for 1 week. Groups were inoculated intradermally in the heel bulb (IDHB) of the right hind foot with 10^3^, 10^4^, 10^5^, or 10^6^ pfu/animal of FMDV A24-P1Deopt, and a control group was inoculated with 10^5^ pfu/animal of FMDV A24 WT. In a second experiment, 16 swine were divided into 4 groups of 4 animals each. Animals were IDHB inoculated as described above with 10^3^, 10^5^, or 10^6^ pfu/animal of FMDV Asia1-P1Deopt or 10^5^ pfu/animal for Asia1 Shamir WT control. For both studies, animals that did not show clinical disease after the initial inoculation were challenged IDHB in the left hind limb with 10^5^ pfu/animal of FMDV homologous WT at 21 days post-inoculation (dpi).

For all swine experiments, following each FMDV inoculation or challenge, temperature and clinical scores were evaluated daily for 7 days by determining the number of toes presenting FMD lesions and the presence of vesicles in the snout and/or mouth. The maximum clinical score considered was 17, and lesions restricted to the site of inoculation were not counted. The % of lymphocytes in the white cell population from whole blood collected in EDTA was measured for the first 7 days using a Hemavet cell counter (Drew Scientific-Erba Diagnostics, Miami Lakes, FL, USA). Samples of serum and nasal swabs were collected on the day of inoculation (baseline) and daily for 7 days after inoculation. After the first week, serum samples were collected weekly to assess the neutralizing antibody responses.

### 2.8. Detection of Virus in Sera and Nasal Swabs

Mice and swine sera and swine nasal swabs were assayed for the presence of virus using plaque assays on BHK-21 cells. Virus titers were expressed as log_10_ plaque-forming units (pfu)/mL of mice serum or tissue culture infectious dose 50 (TCID_50_)/mL of swine serum or nasal swab. The detection level of this assay is 5 pfu/mL. In addition, FMDV RNA in swine sera and nasal swabs was detected using real-time RT-PCR (rRT-PCR) as previously described [28]. Cycle threshold (Ct) values were converted to RNA copies per milliliter using the equation derived from the analysis of serial 10-fold dilutions of in vitro-synthesized FMDV RNA of known concentration and expressed as the genome copy number per mL of serum or nasal swab.

### 2.9. Evaluation of Humoral Immune Response

Neutralizing antibody titers were determined in mice or swine sera through end-point titration according to the method of Kärber [29]. Antibody titers were expressed as the log_10_ value of the reciprocal of the dilution that neutralized 100 TCID_50_ in 50% of the wells. 

### 2.10. Data Analyses

Data handling, analysis, and graphic representations were performed using Prism 5.0 (GraphPad Software, San Diego, CA, USA) or Microsoft Excel (Microsoft, Redmond, WA, USA). Statistical significance was determined using Student’s *t* test.

## 3. Results 

### 3.1. Characterization of FMDV A24 and Asia1 with Synonymous Codon Pair Bias Deoptimization of the P1 Region in Porcine Cells

**Figure 1:**
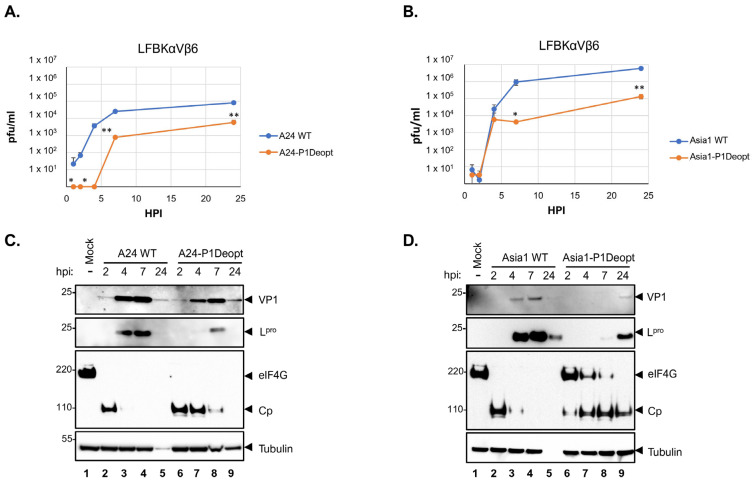
Kinetics of growth of A24 and Asia P1-Deopt FMDV. LF-BKαvβ6 cells were infected with either A24 WT and A24-P1Deopt (**A**) or Asia1 WT and Asia1-P1Deopt (**B**) at a MOI = 5. At various times post-infection (1, 2, 4, 7, and 24 h post-infection (hpi)), virus titers (pfu/mL) in the supernatant were determined using plaque assays in BHK-21 cells. The values are presented as the mean ± standard deviation (SD) of three independent experiments. (**C**,**D**) Western blotting analysis of LF-BKαvβ6 cell lysates infected with either A24 WT and A24-P1Deopt or Asia1 WT and Asia1-P1Deopt. Shown are the representative results from three independent experiments. Tubulin signal serves as a loading control for both C and D. Molecular weight markers’ relative positions are indicated to the left of each panel. Cp stands for cleavage product. Statistical analysis was performed using Student’s t test. *, *p*  <  0.05; **, *p* < 0.005.

We previously demonstrated that FMDV can tolerate sequence deoptimization in the P1 capsid coding region of FMDV serotype A (strain A12), rendering viable virus that is attenuated in mice and swine [21]. To evaluate if this approach could be applied to other FMDV serotypes and strains, the P1 regions of FMDV A24 and FMDV Asia1 Shamir were designed using codon pair bias deoptimization (CPD) with the synthetic attenuated virus engineering (SAVE) method [26] followed by de novo synthesis and cloning into pA_24_Cru infectious clones using the flanking *FseI* and *NheI* restriction enzyme cut sites as previously described [22]. CPD in the P1 region of FMDV A24 resulted in 487 nucleotide substitutions and a 1.40- and 1.27-fold increase in the dinucleotides CpG and UpA, respectively, within the entire FMDV open reading frame (ORF). Similarly, CPD in the P1 coding region of FMDV Asia1 contained 485 nucleotide substitutions, and resulted in a dinucleotide frequency fold increase of 1.42 for CpG and 1.49 for UpA. A24-P1Deopt and Asia1-P1Deopt infectious virus were recovered in BHK-21 cells and purified through sucrose gradient ultracentrifugation. To examine the replication characteristics of the deoptimized viruses, growth curves of the WT and the deoptimized viruses were compared at a multiplicity of infection (MOI) of 5 in the porcine cell line LF-BKαVβ6 (Figure 1A,B). Both A24-P1Deopt and Asia1-P1Deopt exhibited a significant reduction (≥1-log) in viral replication at 7 and 24 hpi when compared to WT viral titers. 

To better understand the reduced titers in the growth curve of deoptimized viruses, we conducted Western blotting analysis on lysates from LF-BKαVβ6 cells infected with either WT or P1Deopt viruses at various time points. This analysis was focused on the expression of viral proteins and known cellular proteins that are targeted for degradation during FMDV replication. As seen in Figure 1C,D, signals for the structural protein VP1 and the non-structural Leader proteinase (L^pro^) were detected at 4 and 7 hpi in samples infected with WT viruses. In contrast, VP1 and L^pro^ signals were reduced during the infection with P1Deopt viruses. Furthermore, in the case of Asia1-P1Deopt infection, L^pro^ and VP1 signals were detected later, mainly at 24 hpi (Figure 1D). During infection with FMDV WT virus, the cleavage of the cellular protein eIF4G is a characteristic of the infection [30,31], starting as early as 2 h after infection. However, the cleavage of eIF4G was altered during the course of infection with the two P1Deopt viruses (A24 and Asia1) as compared to the WT homologous viruses. Furthermore, the eIF4G cleavage profile were distinct depending on the deoptimized FMDV strain. During A24-P1Deopt virus infection, the initial cleavage of eIF4G was not affected, since the disappearance of the full length eIF4G (220 kDa) protein and the appearance of the corresponding cleavage product (Cp) (~120 kDa) were detected during infection at 2 hpi. However, complete loss of the Cp was not observed during infection with A24-P1Deopt until 24 hpi, as compared to 4 hpi for A24 WT infection. Lysates infected with the Asia1-P1Deopt virus suffered from a delay of the initial eIF4G cleavage at 2, 4, and 7 hpi and never demonstrated a complete loss of the eIF4G Cp. Overall, these results suggest that the deoptimization of the P1 region in the FMDV A24 or Asia1 Shamir strains results in viable viruses with a substantial level of attenuation in porcine cells, as well as a slightly different attenuation phenotype in vitro depending on the deoptimized serotype. 

### 3.2. P1 Deoptimization of A24 and Asia1 Results in Attenuation of FMDV in Mice and Confers Protection against WT Homologous Challenge

**Figure 2:**
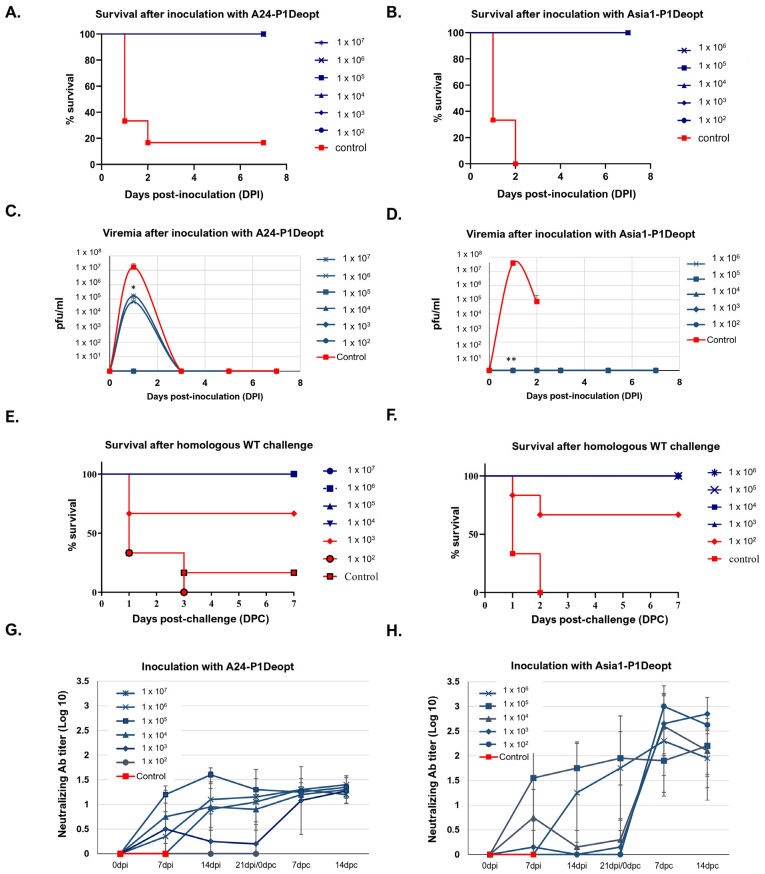
P1 Deoptimized viruses are attenuated in mice. In our experiment, 6- to 7-week-old female C57BL/6 mice (n = 6/group) were subcutaneously (SQ) inoculated in the footpad with either A24-P1Deopt or Asia1-P1Deopt at the indicated doses (pfu/animal). Control groups for each P1Deopt mice experiment included a group inoculated with 1 × 10^5^ pfu/animal of A24 WT or 1 × 10^4^ pfu/animal of Asia1 WT. (**A**,**B**) Survival rates determined daily post-inoculation for 7 days. (**C**,**D**) Virus titers were measured in serum samples collected for 7 days post-inoculation (dpi). (**E**,**F**) Survival rates of group of mice after challenge with either A24 WT (**E**) or Asia1 WT (**F**) determined daily post-challenge for 7 days. (**G**,**H**) FMDV specific antibody neutralizing titers for each mouse experiment were measured in serum samples collected 0, 5, 7, 14, and 21 dpi with deoptimized variants and 7 and 14 days post-challenge (dpc) in all animals that survived the initial inoculation and evaluated using a microtiter neutralization assay on BHK-21 cells. Data are expressed as average ± SD of all animals in each group. Statistical analysis was performed using Student’s *t* test. *, *p*  <  0.05; **, *p* < 0.005.

To characterize the attenuated phenotype of A24-P1Deopt and Asia1-P1Deopt in vivo, we examined their virulence using a mouse model of FMD [32]. Since the FMDV strain Asia1 Shamir had not been characterized in mice previously, preliminary lethality experiments were conducted in groups of inbred C57BL/6 mice (n = 6) using tenfold serial dilutions of the Asia1 Shamir WT virus (10^2^ to 10^6^ pfu/mouse). Mice infected with Asia1 Shamir displayed characteristic clinical signs of FMD, such as lethargy and roughened fur (data not shown) [33], and started to die at 2 days post-inoculation (dpi). A dose of 10^4^ pfu/mouse resulted in 16.67% survival, while a minimum dose of 10^5^ pfu/mouse was required to cause 100% lethality (Supplementary Figure S1A). Mortality was found to be correlated with viremia levels of ≥10^7^ pfu/mL in serum (Supplementary Figure S1B). The virulence of the A24-P1Deopt and Asia1-P1Deopt viruses was determined in groups of mice (n = 6) inoculated with different doses ranging from 10^2^ to 10^7^ pfu/mouse (Figure 2). Clinical signs, survival rate, and the presence of infectious virus in blood were monitored daily for a week after inoculation. Mice in the control groups, inoculated with 10^5^ pfu or 10^4^ pfu of WT FMDV A24 and Asia1, respectively, developed clinical signs of FMD (data not shown), and the majority died within 2 days post-inoculation (dpi). In contrast, none of the mice inoculated with the deoptimized viruses showed clinical signs or died throughout the evaluation period, regardless of the administered dose (Figure 2A,B). The lethality of WT A24 and Asia1 in inoculated mice from the control groups was correlated with viremia levels of ≥10^7^ pfu/mL (Figure 2C,D). However, only mice inoculated with the highest doses of A24-P1Deopt (10^7^ and 10^6^ pfu) developed viremia, but with lower titers (<10^6^ pfu/mL) (Figure 2C), while viremia was undetectable in animals inoculated with Asia1-P1Deopt (Figure 2D). Interestingly, inoculation with any deoptimized viral strains resulted in protection against challenge with the WT parental virus in groups of mice receiving the highest doses (10^4^ pfu and higher for A24-P1Deopt and 10^3^ pfu and higher for Asia1-P1Deopt) (Figure 2E,F). As expected, protection was correlated with the presence of dose-dependent neutralizing antibodies detected as early as 7 dpi (Figure 2G,H). 

### 3.3. Deoptimization of P1 Coding Region Results in Attenuation of FMDVA24 and Asia1 in Swine

**Figure 3:**
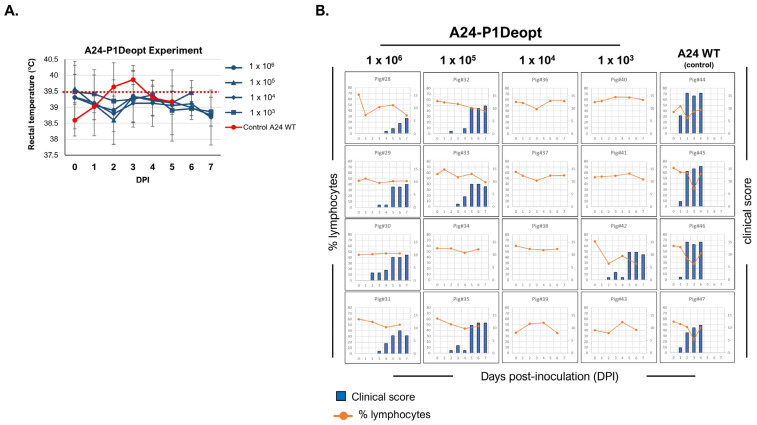
Clinical outcome in swine inoculated with A24-P1Deopt virus. For this experiment, 18–23 kg castrated male Yorkshire swine (n = 4/group) were inoculated with 10^6^ plaque-forming units (pfu) (animals 28 to 31), 10^5^ pfu (animals 32 to 35), 10^4^ pfu (animals 36 to 39), or 10^3^ pfu (animals 40 to 43) of FMDV A24-P1Deopt or 10^5^ pfu of A24 WT (animals 44 to 47) as the control group. Animals were monitored for 7 days and samples of heparinized blood collected every other day. (**A**) Comparison of mean rectal temperatures (±SD) among pigs inoculated with different doses of A24-P1Deopt or A24 WT virus at various times post-inoculation. Temperatures above the dashed line (≥39.5 °C) are considered fever. (**B**) Clinical score (blue bars) and % of lymphocytes (orange line) for each animal are represented.

Building on the observation that P1Deopt viruses were significantly attenuated in mice, we conducted two experiments to investigate whether these strains also exhibited attenuated virulence in swine, which are considered one of the most relevant host species for FMDV. In the first experiment, we examined the virulence of A24-P1Deopt in groups of four pigs inoculated via IDHB with 10^3^, 10^4^, 10^5^, and 10^6^ pfu/animal (Table 1). A control group received 10^5^ pfu/animal of A24 WT (dose previously described to cause clinical signs of FMD in all animals within 1 or 2 days after inoculation [22]). As seen in Figure 3, pigs infected with the WT strain quickly developed symptoms of FMD, including elevated rectal temperatures (≥39.5 °C), lymphopenia and the appearance of vesicular lesions in the feet and/or snout, that were detected as early as 1 dpi and reached a high clinical score (10–17, 17 being the maximum score) by 4 dpi. Although animals inoculated with 10^5^–10^6^ pfu/pig A24-P1Deopt developed lesions, the onset of the disease was delayed (2 or 3 dpi) and the overall severity was reduced compared to animals inoculated with the WT strain (maximum clinical score of 12 observed compared to 17 in WT). Notably, the animals inoculated with deoptimized virus never displayed fever throughout the study, and one animal inoculated with 10^5^ pfu (pig#14) never developed the disease. Interestingly, animals inoculated with 10^4^ or 10^3^ pfu of A24-P1Deopt did not display any clinical signs of the disease, except for one animal (pig#42) in the group that received the lowest dose (10^3^ pfu). This animal developed vesicular lesions at 2 dpi and reached a maximum clinical score of 11 at 5 dpi. 

**Figure 4:**
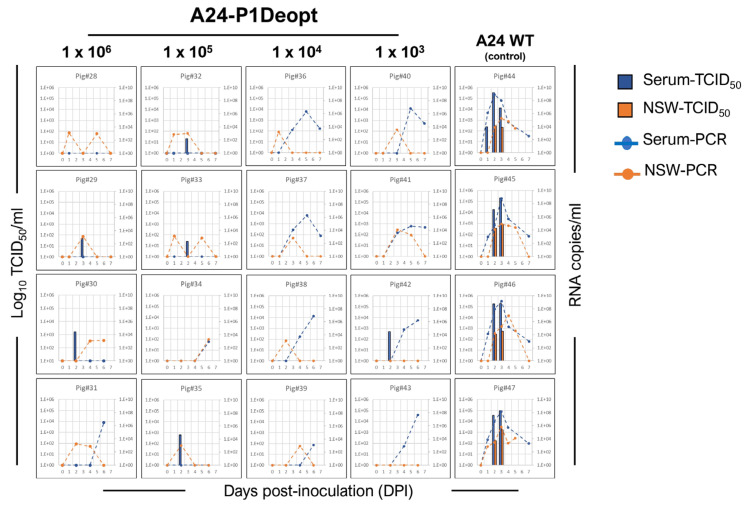
Detection of virus in serum and nasal swabs from animals inoculated with A24-P1Deopt virus. Virus isolation in serum (blue bars) and nasal secretion (orange bars) and presence of viral RNA genome copy numbers per mL of serum (blue dashed line) and nasal secretion (orange dashed line) are shown for each animal at various times post-inoculation.

Serum samples collected from pigs infected with A24 WT consistently showed the presence of viremia, indicated by the detection of viral RNA copies and the presence of infectious virus in the serum, one day before or concurrent with the onset of clinical disease and lymphopenia (Figure 4). Viral loads reached their highest levels (virus isolation: 10^5^ TCID_50_/mL; RT-PCR: 10^8^ RNA copies/mL) by 2–3 dpi. Compared to pigs infected with the WT strain, animals inoculated with high doses of the A24-P1Deopt virus showed significantly lower detection rates and levels of viremia. In some cases, the viremia in these animals could only be detected at much later time points than those observed in the control groups (Figure 4; 10^2^–10^3^ TCID_50_/mL at 2–3 dpi; RT-PCR: 10^6^ RNA copies/mL at 6 dpi). Interestingly, animals inoculated with the lower doses of A24-P1Deopt that did not develop disease had higher values of FMDV RNA copies in the serum (RT-PCR: 10^6^–10^7^ RNA copies/mL) compared to the groups that received higher doses of A24-P1Deopt virus. However, irrespective of the detected FMDV RNA viral loads, virus isolation from the sera of these animals was unsuccessful. In parallel to viremia, animals inoculated with the WT strain had detectable virus in nasal swabs starting at 2 to 3 dpi (10^2^–10^3^ TCID_50_/mL; 10^3^–10^4^ RNA copies/mL). Nasal swab viral loads from animals infected with A24-P1Deopt also ranged between 10^2^ and 10^4^ RNA copies/mL starting at 1 to 3 dpi. However, the detection of infectious virus through virus isolation from these samples was not successful. These findings suggest that A24-P1Deopt FMDV exhibits a substantially lower level of virulence in swine as compared to the WT strain.

**Figure 5:**
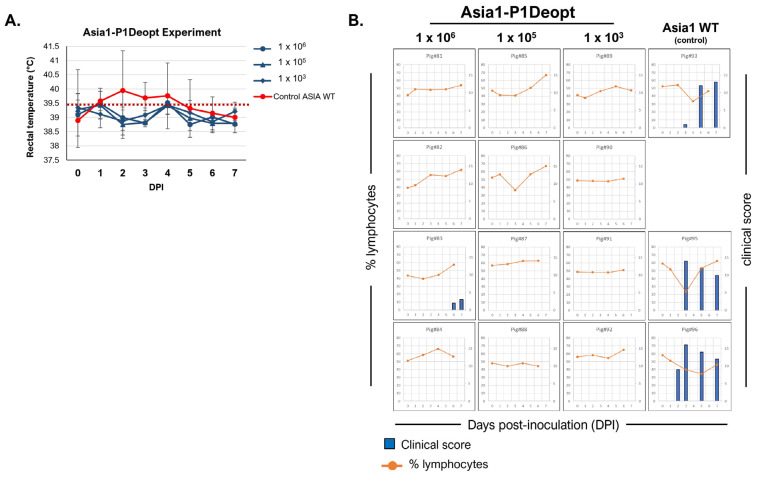
Clinical outcome in swine inoculated with Asia1-P1Deopt virus. For this experiment, 18–23 kg castrated male Yorkshire swine (n = 4/group) were inoculated with 10^6^ plaque-forming units (pfu) (animals 81 to 84), 10^5^ pfu (animals 85 to 88), or 10^3^ pfu (animals 89 to 92) of FMDV Asia-P1Deopt or 10^5^ pfu of Asia1 WT (animals 93, 95, 96) as a control group. Animals were monitored for 7 days and samples of heparinized blood collected every other day. (**A**) Comparison of mean rectal temperatures ± SD among pigs inoculated with different doses of Asia1-P1Deopt or Asia1 WT virus at various times post-inoculation. Temperatures above the dashed line (≥39.5 °C) are considered fever. (**B**) Clinical score (blue bars) and % of lymphocytes (orange line) for each animal are represented.

**Figure 6:**
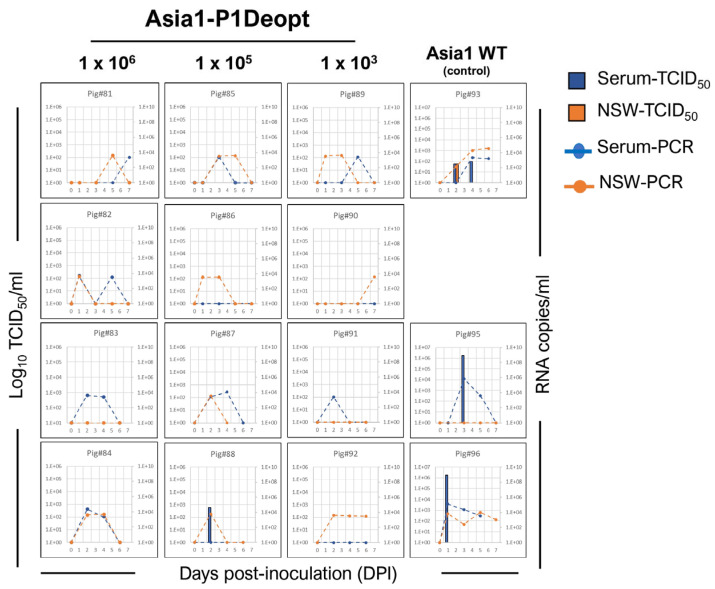
Detection of virus in serum and nasal swabs from animals inoculated with Asia1-P1Deopt virus. Virus isolation in serum (blue bars) and nasal secretion (orange bars) and presence of viral RNA genome copy numbers per mL of serum (blue dashed line) and nasal secretion (orange dashed line) are shown for each animal at various times post-inoculation.

In the second experiment, three groups of four animals each were inoculated IDHB with Asia1-P1Deopt at 10^3^, 10^5^, and 10^6^ pfu/animal, and one group (n = 3) was inoculated with 10^5^ pfu/animal of Asia1 WT as a control [34] (Table 2). As expected, animals inoculated with Asia1 WT showed elevated temperature (Figure 5A), vesicles, and lymphopenia starting 2 or 3 days after inoculation, reaching maximal clinical scores of 13–16 (Figure 5B). Consistently, viremia was detected (10^1^–10^6^ TCID_50_/mL) either a day before or concurrent with the appearance of clinical disease (Figure 6). Only one animal (pig#93) had detectable infectious virus in nasal swabs, with a titer of 5 × 10^1^ TCID_50_/mL at 2 dpi, whereas two out of three animals in the same group showed positive nasal swab samples detected using rRT-PCR. Remarkably, none of the groups inoculated with Asia1-P1Deopt virus developed clinical signs, except for one animal (pig#83) that had a low clinical score (4) at 7 dpi (Figure 5B). Virus isolation was not detected in the sera or nasal swabs of the Asia1-P1Deopt-inoculated animals, except for pig#88, despite detectable RNA viral loads in the range of 10^3^–10^4^ RNA copies/mL (Figure 6). Altogether, these results indicate that the deoptimization of the FMDV P1 region causes consistent viral attenuation in swine independently of the serotype analyzed.

### 3.4. Humoral Response in Swine Inoculated with P1Deopt Attenuated Strains

**Figure 7:**
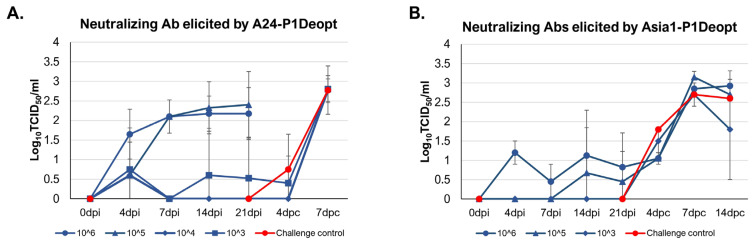
Determination of FMDV neutralizing antibodies in the serum of swine inoculated with deoptimized virus. Presence of FMDV neutralizing antibodies in sera of animals inoculated with different doses of A24-P1Deopt (**A**) or Asia1-P1Deopt (**B**) and after challenge, at the indicated time points after inoculation or challenge, was evaluated using a microtiter neutralization assay on BHK-21 cells. Titers are reported as the log_10_ of the reciprocal of the highest dilution of serum that neutralized the virus in 50% of the wells. Each data point represents the mean ± SD of each group.

To assess the feasibility of A24-P1Deopt and Asia1-P1Deopt as potential LAV candidates against FMD, humoral responses were evaluated at different times after inoculation. As seen in Figure 7, a dose-dependent induction of neutralizing antibodies was detected before animals were challenged. In the A24-P1Deopt experiment, serum showed presence of neutralizing antibodies as early as 4 dpi, although consistent humoral induction over time was mainly observed in animals receiving 10^6^ and 10^5^ pfu/animal with values of ≥2 logs by 14–21 dpi (Figure 7A). In the Asia1-P1Deopt experiment, animals inoculated with higher doses of viruses (10^5^ and 10^6^ pfu/animal) exhibited detectable neutralizing antibodies, albeit with low titers (0.5–1 log) (Figure 7B). To evaluate if the observed humoral response was sufficient to confer protection, animals that did not show clinical disease after the initial inoculation with either A24-P1Deopt (only those inoculated with 10^4^ or 10^3^ pfu/animal) or Asia1-P1Deopt (all animals) were subsequently challenged with their respective homologous WT viruses. Consistent with the low levels of humoral response observed prior to challenge, animals inoculated with the deoptimized A24 or Asia1 WT viruses that remained asymptomatic after the initial inoculation did not exhibit protection upon challenge with the WT homologous strains (Table 1 and Table 2). Nonetheless, anamnestic responses were detected after challenge (7 dpc) and were directly related with clinical disease induced by the WT challenge virus (Figure 7).

## 4. Discussion

We previously demonstrated the feasibility of implementing a codon pair deoptimization (CPD) approach for the generation of an attenuated FMDV strain based on the recoding of the capsid coding region of P1 in FMDV A12 strain [21]. In this study, we employed the same strategy using the SAVE method [26] to recode the capsid (VP4, VP2, VP3, and VP1) coding sequence of FMDV strains A24 and Asia1 Shamir with the aim of extending research to FMDV strains currently used for commercial vaccines. The ability to recode the capsid coding region of FMDV and rapidly clone it on the A_24_Cru backbone IC offers an invaluable strategy to swiftly develop LAVs for unique serotypes [35]. Although the CPD of the P1 region resulted in no changes at the amino acid level, different degrees of attenuation were observed in vitro and in vivo. In particular, the Asia1-P1Deopt virus demonstrated even greater attenuation than the A24-P1Deopt virus. Specifically, we observed an almost 2-log (100 fold) difference in endpoint titers between Asia1-P1Deopt and the Asia 1 Shamir WT strain in vitro, while more than one log (10 fold) difference was observed between A24-P1Deopt and A24 WT. Parallel to the in vitro results, distinct attenuated phenotypes were observed in mice inoculated with P1Deopt viruses. Both the A24-P1Deopt and Asia1-P1Deopt viruses conferred 100% survival regardless of the dose inoculated. However, detectable viremia was observed in mice inoculated with higher doses of the A24-P1Deopt virus, whereas sera in mice inoculated with Asia1-P1Deopt remained negative throughout the study. Furthermore, the observed attenuated phenotypes were maintained in the natural host. While swine inoculated with 10^5^ or 10^6^ pfu of A24-P1Deopt showed clinical signs of FMD and detectable viremia, pigs inoculated with Asia1-P1Deopt remained clinically healthy and free of viremia throughout the study.

Our previous work demonstrated varying degrees of attenuation within a single FMDV serotype based on the targeted coding region and the extent of deoptimization [22]. This phenomenon is not exclusive to FMDV, but has also been observed in other picornaviruses [26]. While the observed differences in attenuation between A24-P1Deopt and Asia1-P1Deopt cannot be solely attributed to the deoptimization strategy, it is important to note that variations in virulence exist among different FMDV WT serotypes and subtypes [36]. However, the underlying mechanisms governing these differences in pathogenicity are not yet well understood [37,38].

A reduction in replicative fitness, as evidenced by smaller plaques sizes and reduced viral titers, was observed in cell culture for both deoptimized viruses, A24-P1Deopt and Asia1-P1Deopt. Interestingly, the Asia1-P1Deopt virus showed a larger decrease in viral fitness (Figure 1), possibly due to its hybrid profile, which consists of the recoded Asia1 P1 region within the A_24_Cru backbone. It is possible that certain viral RNA–capsid interactions that are specific to the A24 serotype may not be optimal in the derived Asia1-P1Deopt virus, thus affecting fitness. This highlights an important feature of viral replication that entails viral RNA interactions for efficient genome packaging and optimal virus assembly. It has been proposed that different putative packaging signals comprised of secondary RNA structures can be found in the P1 region of FMDV and are essential for viral assembly [39]. Consistently, the design of the deoptimized P1 region in A24 and Asia1 purposely excluded regions with predicted stable secondary RNA structures (data not shown), with the objective of minimizing the potential impact on viral assembly. In fact, our results indicate that the recoding of P1Deopt viruses severely impaired the expression of viral proteins (i.e., VP1), suggesting that the attenuation observed may be linked to a step upstream from viral assembly.

The choice of using the Asia1 WT FMDV as a control for comparing to the phenotype of the Asia1-P1Deopt virus in our study may have imposed a limitation, as the presence of the heterologous capsid cloned in the A24cru IC backbone could have contributed to the observed attenuation. However, our decision was based on the desire to evaluate the efficacy of our construct as an LAV candidate against possible circulating strains or serotypes/subtypes currently used for commercial vaccines, such as Asia 1 Shamir [40].

Several diverse hypotheses to explain the underlying molecular mechanisms of the attenuation of CPD viruses have been proposed, including the effect on translation efficiency [41,42,43]. This is based on the understanding that the kinetics of mRNA translation plays a critical role in protein synthesis and folding [44,45], which is governed by the dynamics of ribosome–protein associations, the availability of transfer RNA, and mRNA sequences that determine codon usage, secondary RNA structures, and GC content [44,45,46]. It is then reasonable to speculate that CPD of viral genomes can decrease the flow of translation. Thus far, experiments designed to evaluate the effects of codon deoptimization on the translation rate of picornaviruses have lacked accordance [41,47]. In agreement with the study by Mueller et al. [41], CPD of the most diverse region of FMDV resulted in reduced capsid expression independent of the serotype; however, our results do not differentiate whether CPD affects translation or replication during infection. Unexpectedly, delayed and reduced protein levels of the protease L^pro^ were also detected in cell culture, which resulted in a failure to efficiently cleave eIF4G (Figure 1). This is perplexing, as the L^pro^ coding sequence is located upstream from the P1 region and was not included in the deoptimization strategy. It is possible that a local delay in translation, caused by CPD of the P1 coding sequence, may affect the folding of the nascent protein, including L^pro^, as it exits the ribosome. Improper folding of L^pro^ could potentially impair its ability to cleave itself from the polypeptide chain, which is necessary for downstream viral replication events. Additionally, CPD of the FMDV P1 region or the addition of the FseI cut site used in our cloning strategy may generate certain RNA structures, leading to favorable interactions with other RNA secondary or tertiary structures of the viral genome impacting translation and/or replication kinetics. Previous reports have suggested that secondary RNA structures in FMDV (5′UTR and 3′UTR) participate in long-range interactions to modulate translation and genome replication [48]. Further investigation is required to determine the effects of CPD on viral secondary RNA structures and how this type of recoding influences their functionality in viral translation and replication.

An unintentional increase in the frequency of dinucleotides CpG and UpA due to CPD can stimulate host immune responses by triggering innate immune sensors (i.e., RIG-I, MDA5, etc.), resulting in the activation of antiviral defense mechanisms. In this study, we observed an increase in CpG and UpA in the full open reading frame of both A24-P1Deopt and Asia1-P1Deopt compared to the parental strains; however, further studies are required to confirm this hypothesis since no systemic antiviral activity could be detected in inoculated animals (data not shown).

The ultimate goal of employing deoptimizing technology to create modified attenuated FMDV strains is to produce safe and efficacious vaccines against FMD. However, ensuring safety is a crucial aspect, particularly concerning the potential for recombination between the vaccine strain and circulating viruses. Recombination events could lead to the emergence of revertants, potentially restoring the virulence of modified attenuated FMDV strains. While this particular study did not delve into recombination events, it is worth noting a recent investigation by Spinard et al. [49] that explored the potential for recombination between WT and FMDV CPD strains. Their study found that although recombination could occur between the FMDV CPD and WT strains, the resulting recombinant viruses exhibited much lower fitness as compared to the WT strains, and consistently became extinct. However, additional studies are needed to explore the various scenarios that could arise considering the high diversity FMDV displays.

Evaluation of the efficacy of CPD FMD strains as LAV candidates is critical. Therefore, it is essential to study the immune response induced by deoptimized viruses in vivo. In this study, the mice inoculated with deoptimized strains showed protective levels of neutralizing antibodies in a dose-dependent manner. Specifically, the A24-P1Deopt virus protected mice at doses of 10^4^ pfu or higher, while the Asia1-P1Deopt protected mice at doses of 10^3^ pfu or higher, which is consistent with the previously described protective dose range for A12-P1Deopt [21]. The immunogenicity of the deoptimized viruses was also evaluated in swine, but the results were slightly different from those observed in mice. In the case of A24-P1Deopt, significant levels of neutralizing antibodies were only induced in the animals that received the highest dose of the virus, which also caused disease. Furthermore, animals vaccinated with lower doses of A24-P1Deopt were not protected against homologous WT challenge. In contrast, despite inducing neutralizing antibody levels >1 log and being attenuated in inoculated pigs at a dose of 10^6^ pfu, Asia1-P1Deopt failed to confer protection against challenge with the homologous WT virus. These results are different than those previously described for A12-P1Deopt and A24-P2/P3Deopt or other attenuated viruses such as A12-SAP, an attenuated FMDV strain with a double amino acid substitution in viral L^pro^, for which doses of 10^5^ pfu were completely innocuous in swine and were still able to induce high levels of neutralizing antibodies at 21 dpi (1.8–2.7 Log^10^TCID_50_/mL) [14,21,22]. The lack of induction of a humoral immune response could be partially attributed to the absence of detectable viable virus in serum. However, it is worth noting that previous studies have demonstrated the induction of specific neutralizing antibodies against attenuated FMDV strains without detectable viremia [21]. On the other hand, recent studies of deoptimized vaccine candidates for other viral diseases (i.e., DENV) [50] showed that some promising candidates that induced protective immune responses in mice were too attenuated to confer protection when tested in more relevant species such as rhesus macaques. Clearly, correlations between serum antibody titers and susceptibility to FMDV infection are rather imperfect, and additional studies are needed to identify strong immunologic correlates of protection for further improvement of LAVs.

A key factor in the optimization of the use of attenuated vaccines is the balance between the safety profile and immunogenicity versus retained virulence. Accordingly, if deoptimization in the P1 region for other relevant serotypes, cloned in the A_24_Cru IC backbone described here, is to be pursued in order to have a repertoire of serotypes ready to deploy, further refinement of the deoptimization parameters to “de-attenuate” the strains in some cases or “attenuate more” in other cases, used in combination with other well-established attenuating mutations of FMDV NS proteins [14], might be required. The algorithms used during the design of the deoptimized sequence can modulate the base substitutions accordingly, as has already been demonstrated for several RNA viruses, including other picornaviruses [26]. Likewise, the strategy of combining deoptimization with attenuating point mutations has recently been applied in order to develop a new vaccine platform for poliovirus, which is currently in phase II of clinical trials in adults, toddlers, and infants [51]. Regardless, each serotype attenuated vaccine candidate seed will have to be designed, produced, and tested individually prior to being ready for large-scale development to fine-tune and balance the safety margin.

## 5. Conclusions

In the current study, we examined the versatility of the recoding system by using CPD applied to the P1 capsid coding region of a different FMDV A subtype, A24, or another serotype, Asia1. The modulation of the codon usage of the FMDV P1 region has enabled us to gain further insights into FMDV biology, including viral replication and pathogenesis. Both deoptimized FMDV strains, the A24-P1Deopt and Asia1-P1Deopt strains, showed clear attenuation in vivo, resulting in varying degrees of adaptive immune responses that were influenced by the deoptimized serotype, inoculation dose, and host species. Our findings highlight the potential of CPD in attenuating viral strains across multiple FMDV serotypes/subtypes. However, it is crucial to address certain limitations before fully harnessing this platform for the development of LAVs against FMD. For instance, its development will require a thorough assessment of virulence and the induction of adaptive immunity in the natural host for each new serotype or subtype. This is essential to strike the right balance between attenuation and the induction of protective immunity. Considerations of adding additional mutations or modifying vaccine schedules by boosting will need to be optimized to best elicit protective immunity in an effort to improve vaccination strategies to control FMD. Overall, the use of deoptimization to generate attenuated FMDV strains represents a promising platform in the development of LAVs against FMD. This approach will pave the way for the rapid and long-lasting protection against viral infection, propelling us closer to the ultimate goal of global FMD eradication.

## Figures and Tables

**Table 1:** Clinical performance of swine inoculated with varying doses of FMDV A24-P1Deopt or A24 WT and after challenge with homologous WT A24.

Group Dose ^a^	Clinical Results after Deoptimized Virus Inoculation							Clinical Results after Challenge with WT Homologous Virus					
	Pig #	Clinical Score ^b^	Viremia ^c^	Viremia-PCR ^d^	Shedding Virus ^e^	Shedding RT-PCR ^f^	SN ^g^	Clinical Score ^h^	Viremia ^i^	Viremia-PCR ^j^	Shedding Virus ^k^	Shedding RT-PCR ^l^	SN ^m^
**1 × 10^6^**	50128	4/6	0/0/0	0/0/0	0/0/0	1/1.24 × 10^3^/5	0/2.4						
	50129	3/9	3/4.75 × 10^1^/1	0/0/0	0/0/0	3/1.28 × 10^3^/3	0/1.5						
	50130	2/10	2/1.55 × 10^3^/1	0/0/0	0/0/0	4/1.16 × 10^3^/6	0/3						
	50131	3/9	0/0/0	6/2.96 × 10^6^/6	0/0/0	2/1.74 × 10^3^/4	0/1.8						
**1 × 10^5^**	50132	2/11	3/2.00 × 10^1^/1	0/0/0	0/0/0	1/6.74 × 10^2^/2	0/1.8						
	50133	3/9	3/2.50 × 10^1^/1	0/0/0	0/0/0	1/1.34 × 10^3^/5	0/2.4						
	50134	0/0	0/0/0	6/8.59 × 10^2^/6	0/0/0	6/2.10 × 10^3^/1	0/3.6						
	50135	2/12	2/6.25 × 10^2^/1	0/0/0	0/0/0	2/2.10 × 10^3^/1	0/1.8						
**1 × 10^4^**	50136	0/0	0/0/0	3/3.30 × 10^3^/7	0/0/0	1/6.88 × 10^2^/1	0/0	1/13	1/1.75 × 10^5^/3	0/2.10 × 10^3^/7	2/3.50 × 10^2^/3	2/3.10 × 10^4^/5	0/2.4
	50137	0/0	0/0/0	3/1.20 × 10^4^/7	0/0/0	3/6.88 × 10^2^/1	0/0	1/16	2/4.50 × 10^5/^3	1/6.97 × 10^5^/7	2/2.65 × 10^4^/3	2/7.10 × 10^6^/5	0/2.7
	50138	0/0	0/0/0	4/5.67 × 10^3^/6	0/0/0	2/1.29 × 10^3^/1	0/0	1/14	2/2.15 × 10^5^/3	0/1.92 × 10^3^/7	2/5.75 × 10^2^/3	3/4.92 × 10^4^/5	0/3
	50139	0/0	0/0/0	6/1.28 × 10^3^/6	0/0/0	4/1.27 × 10^3^/1	0/0	1/17	2/2.28 × 10^5/^3	1/4.40 × 10^1^/7	2/7.75 × 10^2^/3	1/4.80 × 10^2^/5	0/3
**1 × 10^3^**	50140	0/0	0/0/0	5/6.50 × 10^6^/7	0/0/0	3/3.77 × 10^3^/1	0/0	2/16	2/3.70 × 10^5/^3	0/5.23 × 10^2^/7	2/3.75 × 10^3^/3	1/7.00 × 10^2^/5	0/3
	50141	0/0	0/0/0	3/3.92 × 10^3^/7	0/0/0	3/1.21 × 10^4^/2	0/0	2/13	0/0/0	1/5.40 × 10^3^/7	2/7.00 × 10^3^/3	2/4.44 × 10^4^/5	1.2/3
	50142	2/11	2/5.0 × 10^2^/1	4/6.43 × 10^4^/6	0/0/0	0/0/0	0/2.1						
	50143	0/0	0/0/0	4/7.21 × 10^2^/6	0/0/0	0/0/0	0/0	1/14	1/4.00 × 10^5^/3	0/2.26 × 10^3^/7	2/5.50 × 10^3^/3	1/1.08 × 10^3^/5	0/2.4
**Control**	50144	1/16	1/3.40 × 10^5/^3	2/1.33 × 10^6^/7	2/3.00 × 10^2^/3	2/8.23 × 10^3^/5	1.8/2.4						
	50145	1/16	2/2.02 × 10^5/^3	2/9.26 × 10^2^/7	2/8.75 × 10^2^/3	2/1.30 × 10^4^/5	0/2.1						
	50146	1/15	2/1.85 × 10^5^/3	2/1.42 × 10^4^/7	2/5.50 × 10^2^/3	2/1.70 × 10^4^/5	1.2/3.3						
	50147	1/11	2/9.50 × 10^4^/3	2/8.07 × 10^3^/7	2/1.68 × 10^3^/3	1/6.90 × 10^2^/5	0/3.3						

^a^ Dose of inoculum per animal expressed as number of pfu in a total volume of 0.4 mL. ^b^ Days post inoculation (dpi) first signs of lesions are detected/highest lesion score achieved throughout the entire experiment. ^c^ First dpi that viremia was detected using virus isolation techniques; maximum amount of viremia in TCID_50_/mL detected in sera samples; and the duration (days) of viremia. ^d^ First dpi that viremia was detected using Real Time-PCR; maximum amount of viremia in genome copy numbers (GCN)/mL detected in sera samples; and the duration (days) of viremia. ^e^ First dpi that shedding virus was detected using virus isolation techniques; maximum amount of shedding virus in TCID_50_/mL detected in nasal swab samples; and the duration (days) of shedding. ^f^ First dpi that shedding virus was detected using Real Time-PCR; maximum amount of shedding virus in GCN/mL detected in nasal swab samples; and the duration (days) of shedding. ^g^ SN = serum neutralizing antibody response reported as Log_10_ of TCID_50_ at 0 and 21 dpi, respectively. ^h^ Days post challenge (dpc) first signs of lesions are detected/highest lesion score achieved throughout the entire experiment. ^i^ First dpc that viremia was detected using virus isolation techniques; maximum amount of viremia in TCID_50_/_mL_ detected in sera samples; and the duration (days) of viremia. ^j^ First dpc that viremia was detected using Real Time-PCR; maximum amount of viremia in GCN/mL detected in sera samples; and the duration (days) of viremia. ^k^ First dpc that shedding virus was detected using virus isolation techniques; maximum amount of shedding virus in TCID_50_/mL detected in nasal swab samples; and the duration (days) of shedding. ^l^ First dpc that shedding virus was detected using Real Time-PCR; maximum amount of shedding virus in GCN/mL detected in nasal swab samples; and the duration (days) of shedding. ^m^ SN = serum neutralizing antibody response reported as Log_10_ of TCID_50_ at 0 and 21 dpc, respectively. Grey shaded cells correspond to animals that were not challenged.

**Table 2:** Clinical performance of swine inoculated with varying doses of FMDV Asia1-P1Deopt or Asia1 and after challenge with homologous WT Asia1.

Group Dose ^a^	Clinical Results after Deoptimized Virus Inoculation							Clinical Results after challenge with WT Homologous Virus					
	Pig #	Clinical Score ^b^	Viremia ^c^	Viremia-PCR ^d^	Shedding Virus ^e^	Shedding RT-PCR ^f^	SN ^g^	Clinical Score ^h^	Viremia ^i^	Viremia-PCR ^j^	Shedding Virus ^k^	Shedding RT-PCR ^l^	SN ^m^
**1 × 10^6^**	52481	0/0	0/0/0	7/2.53 × 10^3^/1	0/0/0	5/4.42 × 10^3^/1	0/0	3/16	2/5.62 × 10^2^/2	2/5.67 × 10^3^/5	4/1.00 × 10^3^/1	4/1.66 × 10^4^/3	0/3.3
	52482	0/0	0/0/0	1/4.99 × 10^3^/1	0/0/0	1/3.70 × 10^3^/1	0/0	3/17	2/1.78 × 10^3^/1	2/4.04 × 10^4^/5	0/0/0	6/2.3 × 10^3^/1	0/2.7
	52483	2/3	0/0/0	1/4.28 × 10^3^/5	0/0/0	0/0/0	0/1.2	5/7	1/3.16 × 10^2^/3	3/9.15 × 10^3^/5	3/1.78 × 10^2^/1	1/2.11 × 10^4^/5	1.2/3.3
	52484	0/0	0/0/0	2/2.32 × 10^4^/4	0/0/0	2/4.07 × 10^3^/3	0/2.1	7/1	5/3.16 × 10^2^/1	0/0/0	0/0/0	1/8.73 × 10^3^/7	2.1/2.4
**1 × 10^5^**	52485	0/0	0/0/0	3/2.65 × 10^3^/1	0/0/0	3/3.61 × 10^3^/2	0/0	7/1	4/1.00 × 10^2^/1	6/2.29 × 10^3^/1	0/0/0	2/7.78 × 10^4^/5	0/2.4
	52486	0/0	0/0/0	0/0/0	0/0/0	1/3.58 × 10^3^/3	0/0	3/15	2/1.00 × 10^4^/1	2/2.06 × 10^4^/5	0/0/0	4/3.85 × 10^1^/1	0/2.7
	52487	0/0	0/0/0	2/2.84 × 10^3^/4	0/0/0	2/3.78 × 10^3^/1	0/1.8	0/0	0/0/0	1/1.92 × 10^4^/5	0/0/0	1/5.37 × 10^4^/7	1.8/3
	52488	0/0	0/0/0	2/6.08 × 10^3^/1	0/0/0	2/6.08 × 10^3^/1	0/0	3/16	3/1.00 × 10^7^/6	3/1.64 × 10^6^/3	3/1.76 × 10^2^/1	1/9.16 × 10^4^/7	0/3.3
**1 × 10^3^**	52489	0/0	0/0/0	5/3.02 × 10^3^/1	0/0/0	1/3.99 × 10^3^/1	0/0	2/17	2/1.78 × 10^6^/1	2/3.1 × 10^6^/5	2/1.78 × 10^4^/3	2/3.39 × 10^4^/5	0/1.8
	52490	0/0	0/0/0	0/0/0	0/0/0	7/3.94 × 10^3^/1	0/0	2/12	2/1.00 × 10^7/^2	2/8.72 × 10^5/^5	2/1.00 × 10^2^/1	2/2.7 × 10^3^/1	0/2.4
	52491	0/0	0/0/0	2/2.30 × 10^3^/1	0/0/0	0/0/0	0/0	2/17	3/3.16 × 10^5^/5	3/3.16 × 10^5^/3	3/3.16 × 10^2^/1	1/5.37 × 10^4^/7	0/2.4
	52492	0/0	0/0/0	0/0/0	0/0/0	2/4.32 × 10^3^/5	0/0	2/17	3/5.62 × 10^2/^1	3/7.50 × 10^5/^3	3/1.78 × 10^2^/1	1/7.57 × 10^4^/7	0/3
**Control**	52493	3/13	2/1.00 × 10^2^/2	4/2.37 × 10^3^/3	2/5.62 × 10^1^/1	2/4.04 × 10^4^/5	0/2.7						
	52495	3/14	3/1.78 × 10^6/^1	3/6.25 × 10^5/^3	0/0/0	0/0/0	0/2.4						
	52496	2/16	1/1.78 × 10^6/^1	1/1.25 × 10^5/^5	0/0/0	1/1.01 × 10^4^/7	0/2.7						

^a^ Dose of inoculum per animal expressed as number of pfu in a total volume of 0.4 mL. ^b^ Days post inoculation (dpi) first signs of lesions are detected/highest lesion score achieved throughout the entire experiment. ^c^ First dpi that viremia was detected using virus isolation techniques; maximum amount of viremia in TCID_50_/mL detected in sera samples; and the duration (days) of viremia. ^d^ First dpi that viremia was detected using Real Time-PCR; maximum amount of viremia in genome copy numbers (GCN)/mL detected in sera samples; and the duration (days) of viremia. ^e^ First dpi that shedding virus was detected using virus isolation techniques; maximum amount of shedding virus in TCID_50_/mL detected in nasal swab samples; and the duration (days) of shedding. ^f^ First dpi that shedding virus was detected using Real Time-PCR; maximum amount of shedding virus in GCN/mL detected in nasal swab samples; and the duration (days) of shedding. ^g^ SN = serum neutralizing antibody response reported as Log_10_ of TCID_50_ at 0 and 21 dpi, respectively. ^h^ Days post challenge (dpc) first signs of lesions are detected/highest lesion score achieved throughout the entire experiment. ^i^ First dpc that viremia was detected using virus isolation techniques; maximum amount of viremia in TCID_50_/mL detected in sera samples; and the duration (days) of viremia. ^j^ First dpc that viremia was detected using Real Time-PCR; maximum amount of viremia in GCN/mL detected in sera samples; and the duration (days) of viremia. ^k^ First dpc that shedding virus was detected using virus isolation techniques; maximum amount of shedding virus in TCID_50_/mL detected in nasal swab samples; and the duration (days) of shedding. ^l^ First dpc that shedding virus was detected using Real Time-PCR; maximum amount of shedding virus in GCN/mL detected in nasal swab samples; and the duration (days) of shedding. ^m^ SN = serum neutralizing antibody response reported as Log_10_ of TCID_50_ at 0 and 14 dpc, respectively. Grey shaded cells correspond with animals that were not challenged.

## Data Availability

Not applicable.

## Supplementary Materials

The following supporting information can be downloaded at: https://www.mdpi.com/article/10.3390/v15061332/s1, Figure S1: Virulence of Asia1 Shamir WT virus in mice.
