# Supplementary material for: Deoptimization of FMDV P1 Region Results in Robust Serotype-Independent Viral Attenuation
Source: Viruses. 2023 Jun 6;15(6):1332. doi: 10.3390/v15061332 (PMC10301631; doi:10.3390/v15061332)
Supplement: Supplementary file 1 [file viruses-15-01332-s001.zip › viruses-2376590-supplementary.pdf]

A.

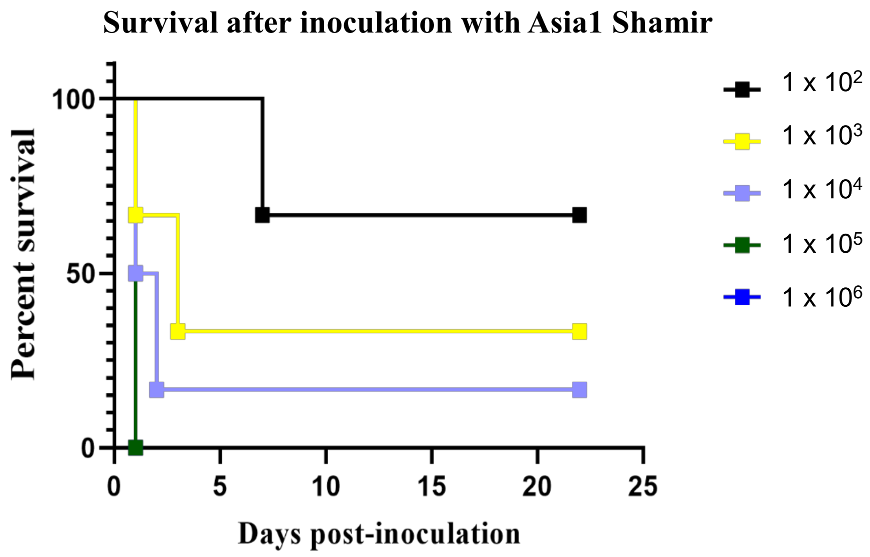

B.

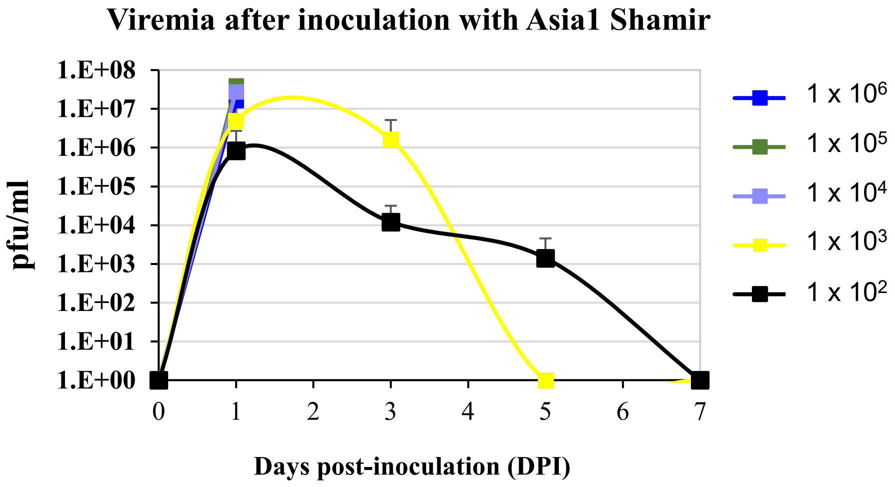

Supplemental Figure S1: Virulence of Asia1 Shamir WT virus in mice. 6 to 7 weeks old female C57BL/6 mice (n = 6/group) were subcutaneously (SQ) inoculated in the footpad with FMDV Asia1 Shamir WT at the indicated doses (pfu/animal). (A) Survival rates determined daily post inoculation (dpi). (B) Virus titers were measured by plaque assay in serum samples collected for 7 dpi and expressed as plaque forming units (pfu)/ ml of serum. Data are expressed as average ± SD of all animals in each group.
